# Acute Modulation of Segmental Angular Momentum by Loaded Whole-Body Vibration During Baseball Batting in Adolescent Athletes

**DOI:** 10.3390/life16101589

**Published:** 2026-09-23

**Authors:** Chao-Fu Chen, Lin Zhu, Jie Meng, Jing-Yi Chen, Yan-Qing Shen

**Affiliations:** 1 School of Physical Education and Sport, Henan University, Kaifeng 475001, China; chenchaofu@henu.edu.cn (C.-F.C.); 251005@hamu.edu.cn (J.M.); 2 Graduate Institute of Sport Coaching Science, Chinese Culture University, Taipei 11114, Taiwan; 3 Physical Education College, Zhengzhou University of Industrial Technology, Zhengzhou 451100, China; zhulin5269@gmail.com; 4 School of Rehabiltation, Henan Medical University, Xinxiang 453003, China; 5 Faculty of Health, University of Canterbury, Christchurch 4800, New Zealand; jingyi.chen@pg.canterbury.ac.nz

**Keywords:** whole-body vibration, baseball batting, angular momentum, kinetic chain, biomechanics, adolescent athletes, external loading

## Abstract

Introduction: Baseball batting requires rapid coordination of multiple body segments as the movement progresses from preparatory loading to trunk rotation and bat–ball contact. Although whole-body vibration (WBV) has been used as a short-duration conditioning method, little is known about how vibration combined with different external loads alters segmental rotational behavior during baseball batting, particularly in adolescent athletes. This study examined phase-specific changes in segmental angular momentum following unloaded and loaded WBV conditions. Methods: Thirty-two national-level adolescent male baseball players were randomly allocated to four conditions: Non-Vibration (Non-Vib), unloaded WBV (Vib), 30% one-repetition maximum combined with WBV (30% 1RM + WBV), and 50%1RM combined with WBV (50% 1RM + WBV). Participants completed a 60-s partial-squat heel-raise conditioning task under their assigned condition, followed immediately by three maximal-effort batting trials. Vibration conditions were performed at 50 Hz with a displacement amplitude of 2 mm. Three-dimensional motion data were obtained using the Kwon3D system, and segmental angular momentum was evaluated during the backswing, hip rotation initiation, and bat–ball contact phases. Results: Intervention-related differences were concentrated in the early phases of the swing. During the backswing, significant group effects were observed for left-shank *Z*-axis, right-upper-arm *Z*-axis, right-forearm *Z*-axis, trunk *Y*-axis, and left-forearm *Y*-axis angular momentum (all *p* < 0.05), with effect sizes ranging from η^2^ = 0.297 to 0.399. Most significant post hoc comparisons involved the 30% 1RM + WBV condition. During hip rotation initiation, trunk *Z*-axis angular momentum differed significantly between the 30% 1RM + WBV and 50% 1RM + WBV conditions, while left-thigh *Z*-axis angular momentum was greater in the 30% 1RM + WBV condition than in the unloaded WBV condition (both *p* < 0.05). No significant between-group differences were detected for segmental or whole-body angular momentum during the bat–ball contact phase. Conclusion: WBV combined with external loading produced phase- and segment-specific alterations in angular momentum during adolescent baseball batting. The 30% 1RM + WBV condition showed the most consistent differences, particularly during the backswing and hip rotation initiation phases, whereas increasing the load to 50% 1RM did not produce systematically greater responses. These findings suggest that moderate-load WBV may modify the organization of segmental rotational motion during the preparatory and early acceleration phases of batting. Further research is needed to determine whether these acute biomechanical changes translate into improvements in batting performance.

## 1. Introduction

Baseball batting is a rapid multisegmental action in which effective performance depends on the coordinated motion of the lower extremities, pelvis, trunk, upper extremities, and bat [1]. Rather than being generated by a single joint or body segment, the swing develops through a sequence of preparatory and rotational actions that progressively organize the body for bat acceleration and ball contact. Lower-extremity support, pelvic and trunk rotation, separation between proximal segments, and the timing of upper-extremity motion have all been recognized as important biomechanical components of successful batting [2]. At the performance level, bat velocity, batted-ball exit velocity, and time to contact are commonly used to characterize the effectiveness of the swing [3]. Accordingly, interventions capable of acutely modifying the mechanical organization of the batting movement may have potential value within pre-activity preparation. Warm-up strategies are commonly used before explosive sports tasks to increase readiness for subsequent high-velocity movements. Dynamic preparation may influence muscle temperature, joint mobility, contraction characteristics, and the ability to rapidly coordinate forceful actions [4]. However, baseball batting imposes demands that extend beyond general increases in strength or range of motion. The movement requires the athlete to organize multiple body segments within a very short time, particularly during the transition from preparatory loading to rapid axial rotation. Therefore, an effective pre-activity intervention for batting should ideally influence not only general physical readiness but also the mechanical behavior of the segments involved in the batting kinetic chain.

Whole-body vibration (WBV) has been increasingly incorporated into strength and conditioning settings as a short-duration conditioning stimulus [5]. Mechanical oscillations generated by WBV are transmitted through the body and may alter sensory input, muscle-tendon behavior, and neuromuscular responses during and immediately after exposure [6]. Within this context, WBV may be considered a specialized pre-activity warm-up or conditioning stimulus rather than merely an isolated exercise modality. One proposed mechanism underlying its acute neuromuscular effects involves vibration-induced activation of muscle spindles and Ia afferent pathways, which may increase α-motor-neuron excitability through mechanisms related to the tonic vibration reflex. Such responses could transiently alter lower-extremity neuromuscular behavior and, through the multisegmental kinetic chain, influence the organization of subsequent sport-specific movements. However, these mechanisms were not directly measured in the present study and therefore remain theoretical explanations rather than demonstrated physiological responses [6]. Previous studies have reported acute changes in strength-, power-, flexibility-, and movement-related outcomes following appropriately prescribed vibration protocols [7]. Nevertheless, the magnitude and direction of these responses appear to depend on several factors, including vibration frequency, displacement amplitude, body position, exposure duration, external resistance, and the timing of the subsequent performance task. Acute WBV studies have frequently used vibration frequencies between approximately 30 and 50 Hz and relatively small displacement amplitudes, with post-intervention effects often assessed within the first several minutes after exposure [8,9,10]. Although such protocols have been investigated extensively in jumping, squatting, and other lower-extremity tasks, their relevance to complex sport-specific skills remains less clear. This distinction is important because an improvement in a relatively simple explosive task cannot automatically be assumed to transfer to a coordinated striking movement. Furthermore, much of the existing evidence has been obtained from adult participants, whereas adolescent athletes may differ in strength, maturation, fatigue tolerance, and neuromuscular coordination. Thus, the acute biomechanical response of adolescent baseball players to WBV requires specific investigation rather than direct extrapolation from adult or general athletic populations. The potential relevance of WBV to baseball batting may be better understood from a multisegmental biomechanical perspective. During the swing, the athlete first establishes a preparatory configuration, then initiates lower-body and proximal rotation before accelerating the trunk and upper extremities toward the ball. Effective batting therefore depends on the coordinated redistribution of mechanical motion across successive body segments [11]. Within this process, segmental angular momentum provides a useful means of describing the magnitude and direction of rotational motion in individual body segments. Examining angular momentum across different phases of the swing may therefore reveal intervention-related changes that would not be apparent from isolated joint angles or velocities alone. This phase-based approach is particularly relevant when evaluating an acute conditioning intervention. A stimulus may alter the preparation of the lower extremities or trunk early in the swing without producing an equivalent difference at the instant of bat–ball contact. Consequently, averaging biomechanical variables across the entire movement may obscure meaningful phase-specific responses. Dividing the batting sequence into the backswing phase, hip rotation initiation phase, and bat–ball contact phase provides a more specific framework for examining how segmental angular momentum is organized as the batter progresses from preparation to forward rotation and ultimately to impact. External loading may further modify the acute response to WBV. Adding resistance changes the mechanical demand placed on the athlete during the conditioning activity and may therefore influence the subsequent balance between stimulation and fatigue [12]. A relatively moderate load may provide sufficient mechanical challenge while allowing rapid movement to be maintained, whereas a heavier load may impose greater mechanical demand and potentially influence the subsequent high-speed task differently. However, whether increasing the external load during WBV produces progressively greater effects on baseball batting mechanics has not been established. In particular, little is known about whether unloaded WBV and loaded WBV produce different patterns of segmental angular momentum during specific phases of the baseball swing.

The limited evidence is especially relevant for baseball-specific research. Most WBV studies have emphasized general measures such as vertical jumping, lower-extremity strength, or isolated power performance rather than the biomechanical organization of a complex striking task [5,6,7,8,9,10,11,12]. As a result, it remains unclear whether WBV alters the distribution of rotational motion across the lower extremities, trunk, and upper extremities during batting, or whether any such effects depend on the magnitude of external loading. Clarifying these responses may provide a more biomechanically specific understanding of how short-duration vibration-based conditioning influences a sport-specific kinetic chain. Therefore, the purpose of the present study was to examine the acute effects of WBV combined with different external loading levels on segmental angular momentum during baseball batting in adolescent athletes. Four experimental conditions were compared: Non-Vibration (Non-Vib), Vibration (Vib), 30% one-repetition maximum combined with WBV (30%1RM+WBV), and 50% one-repetition maximum combined with WBV (50%1RM+WBV). Angular momentum was examined across the backswing, hip rotation initiation, and bat–ball contact phases to determine whether the intervention effects were phase- and segment-specific. It was hypothesized that WBV-based conditions would modify segmental angular momentum relative to the Non-Vib condition, particularly during the preparatory and early rotational phases of the swing. It was further hypothesized that combining WBV with external resistance would produce load-dependent differences in lower-extremity, trunk, and upper-extremity angular momentum; however, because the balance between acute stimulation and mechanical demand may vary according to loading magnitude, a strictly linear increase in biomechanical response with increasing load was not assumed.

## 2. Materials and Methods

### 2.1. Participants

Thirty-two national-level adolescent male baseball players volunteered to participate in the study (age: 15.8 ± 0.6 years; height: 179.3 ± 5.4 cm; body mass: 70.8 ± 6.9 kg). The available national-level training cohort consisted of male athletes; accordingly, the present findings should not be generalized directly to female adolescent baseball or softball players. Participants were randomly allocated to four experimental groups: Non-Vibration (Non-Vib; *n* = 8), Vibration (Vib; *n* = 8), 30% 1RM plus whole-body vibration (30% 1RM + WBV; *n* = 8), and 50% 1RM plus whole-body vibration (50% 1RM + WBV; *n* = 8). An a priori power analysis was not performed because recruitment was constrained by the availability of national-level adolescent athletes and the logistical demands of controlled three-dimensional biomechanical testing. The relatively small group size should therefore be considered when interpreting the findings. All batting trials were performed using each participant’s habitual batting side. Baseline age, height, body mass, body mass index, and years of sport participation were compared by one-way analysis of variance, and no significant between-group differences were identified (all *p* > 0.05). The study protocol was approved by the local university Academic Ethics Committee and was conducted in accordance with the Declaration of Helsinki and its subsequent amendments. Written informed consent was obtained from every participant and from a legal guardian before testing. A CONSORT-style flow diagram of participant recruitment, randomization, intervention allocation, follow-up, and final analysis is presented in Figure 1.

### 2.2. Study Design and Experimental Procedures

A parallel-group randomized controlled design was used to examine the immediate effects of WBV combined with different external loading levels on baseball batting biomechanics. Testing was completed across two laboratory visits separated by 72 h. The first visit was used to obtain baseline batting measurements, whereas the second visit consisted of the assigned conditioning intervention followed immediately by post-intervention batting assessment. The 72-h interval was adopted to reduce the influence of residual fatigue, short-term motor-learning effects, and carry-over neuromuscular responses. To improve measurement consistency, both visits were scheduled at approximately the same time of day and were conducted under standardized indoor conditions. Participants were asked to refrain from strenuous exercise for 24 h before each visit and to maintain their habitual diet and sleep routines throughout the experimental period.

#### 2.2.1. One-Repetition Maximum Assessment

Before the loaded WBV conditions were administered, each participant completed a deep-squat one-repetition maximum (1RM) assessment. Testing was supervised by experienced national-level baseball coaches, with spotters positioned beside the participant for safety. Following a standardized warm-up and several familiarization repetitions with light resistance, the external load was progressively increased until only one technically valid repetition could be completed.

A repetition was accepted when the participant maintained appropriate trunk control, reached the prescribed squat depth, and returned to the upright position without external assistance. Recovery intervals of 3–5 min were provided between maximal attempts to limit fatigue. The greatest successfully completed load was recorded as the participant’s deep-squat 1RM and was subsequently used to calculate individualized loads corresponding to 30% and 50% of 1RM.

#### 2.2.2. Whole-Body Vibration Conditioning

The 30% and 50% 1RM loads were selected to represent two practically distinct submaximal loading levels during WBV conditioning. The 30%1RM condition was intended to provide a moderate external stimulus while allowing the partial-squat heel-raise task to be performed without excessive mechanical demand, whereas the 50%1RM condition represented a higher submaximal load that could provide a stronger conditioning stimulus but potentially increase acute fatigue. These two loading levels were therefore selected to examine whether the biomechanical response to loaded WBV followed a progressively load-dependent pattern.

The conditioning task consisted of a 60-s partial-squat heel-raise exercise performed under the condition assigned to each group. Immediately after the conditioning task, participants completed three maximal-effort batting trials. The trial producing the highest batted-ball exit velocity was retained for the subsequent biomechanical analysis.

During the partial-squat heel raise, the knee joint angle was maintained at approximately 120°. The target position was established during familiarization using a goniometer rather than visual estimation alone, and the research staff monitored posture throughout the 60-s intervention. Verbal correction was provided when necessary to maintain the prescribed position as consistently as possible [13]. Standardizing the lower-limb posture was intended to reduce between-participant variation in vibration transmission and to limit unnecessary mechanical loading during WBV exposure [1].

For all vibration conditions, the platform was operated at 50 Hz with a displacement amplitude of 2 mm. Identical vibration settings were used in the Vib, 30%1RM+WBV, and 50%1RM+WBV groups so that the principal experimental contrast among the WBV conditions was the magnitude of external loading. The selected settings were within ranges previously used to elicit acute neuromuscular and explosive-performance responses (approximately 30–50 Hz and 2–5 mm) [8,9,10]. The relatively small amplitude was chosen to reduce excessive mechanical stress and discomfort in adolescent athletes.

#### 2.2.3. Batting Task and Laboratory Setup

Before formal data collection, the batting area and all measurement devices were arranged and calibrated. Standardized batter’s boxes were marked on both sides of home plate. Each box measured 1.82 m in length and 1.22 m in width, and its inner boundary was positioned 0.15 m from the edge of home plate. Participants completed familiarization swings before the formal trials so that they were accustomed to the testing area, batting tee, and required task.

Batting was performed from a stationary tee to control ball position across trials. Following the procedure described by Reyes et al. [14], tee height was individualized from the vertical height of the greater trochanter of the lead leg while the participant adopted the habitual ready stance. Once established, the same tee height was used for all pre- and post-intervention trials for that participant. The same type of regulation baseball was used throughout testing to minimize variability in bat–ball contact characteristics.

A calibrated capture volume measuring approximately 2.0 × 1.5 × 2.0 m was established around the hitting area. The global laboratory axes were defined by anatomical direction: the *X*-axis represented the mediolateral (left–right) direction, the *Y*-axis represented the anteroposterior (forward–backward) direction, and the *Z*-axis represented the vertical (up–down) direction. Three-dimensional calibration was performed using 12 control points with known spatial coordinates [12,13,14,15].

#### 2.2.4. Motion Capture and Ball-Velocity Measurement

A 14-segment biomechanical model was used for three-dimensional reconstruction. The model comprised the head, trunk, and bilateral upper arms, forearms, hands, thighs, shanks, and feet. Reflective markers were attached to standardized anatomical landmarks, including the head, bilateral acromion processes, elbows, wrists, hand regions, greater trochanters, knees, ankles, heels, and toe regions. Marker trajectories were used to reconstruct segmental motion and to calculate joint- and segment-level kinematic variables in the Kwon3D motion-analysis system [12,13,14,15].

A synchronized high-speed camera system was arranged around the calibrated capture volume to obtain complementary views of the batting motion. The cameras were positioned approximately 4 m from the geometric center of the calibrated space and recorded at 240 Hz with a shutter speed of 1/1000 s. Camera alignment, focus, white balance, exposure, and temporal synchronization were checked before formal acquisition and were kept unchanged during testing.

Batted-ball exit velocity was recorded with a radar speed gun positioned along the projected ball-flight direction and approximately level with the hitting plane. The device was located outside the expected travel paths of the bat and ball to avoid interference with the task [16,17,18,19]. The experimental arrangement, including the camera positions, batting area, tee, and radar-gun location, is illustrated in Figure 2.

For each testing condition, participants performed three maximal-effort swings. Exit velocity was recorded for every valid trial, and the swing with the greatest exit velocity was selected as the representative trial. The corresponding synchronized high-speed recordings were imported into Kwon3D for digitization, three-dimensional reconstruction, and calculation of the kinematic variables used in the statistical analyses. Importantly, batted-ball exit velocity was used solely to identify the representative maximal-effort trial and was not treated as a dependent outcome in the between-group statistical analyses. Bat displacement velocity, elbow angular velocity, knee angular velocity, and pelvic displacement velocity were not included as outcome variables in the current analysis; the inferential analyses reported in Table 1, Table 2 and Table 3 were restricted to segmental angular momentum.

### 2.3. Definition of Batting Phases and Key Events

To standardize biomechanical data extraction, the batting motion was partitioned using four identifiable key events and three continuous phases (Figure 3) [17,18,19,20,21]. The lead-foot lift event was defined as the first frame in which the vertical velocity of the lead heel changed from zero to a positive value. The center-of-mass zero-crossing event was identified after lead-foot lift and before hip rotation as the frame in which the anteroposterior velocity of the body center-of-mass reference, operationalized from the pelvic center, crossed zero or reached the value closest to zero. Hip-rotation onset was defined as the first frame in which the pelvis initiated rapid rotation toward the hitting direction. Bat–ball contact was defined as the first frame showing physical contact between the bat and the ball.

The Backswing Phase extended from lead-foot lift to the center-of-mass zero-crossing event and represented the preparatory loading period. The Hip Rotation Initiation Phase extended from the center-of-mass zero-crossing event to hip-rotation onset and represented the transition from loading to proximal kinetic-chain acceleration. The Bat–Ball Contact Phase extended from hip-rotation onset to bat–ball contact and represented the final acceleration and energy-transfer period preceding impact.

The center-of-mass zero-crossing event corresponds to the previously described Center-of-Gravity Zero Moment. In the revised phase-based framework, this event has not been removed; rather, it is retained as a key temporal boundary separating the Backswing Phase from the Hip Rotation Initiation Phase instead of being analyzed as an independent outcome time point. This restructuring permits angular momentum to be evaluated over biomechanically meaningful continuous phases while preserving the original event as the transition between preparatory loading and the initiation of proximal rotation.

All events were identified frame-by-frame from the synchronized high-speed recordings and the reconstructed three-dimensional kinematic data. Three national-level baseball coaches independently reviewed the event locations, and disagreements were resolved by discussion until consensus was reached. Inter-rater reliability was not quantified and should therefore be considered when interpreting event-based analyses.

Raw three-dimensional marker coordinates were smoothed using a fourth-order Butterworth low-pass filter with a cutoff frequency of 6 Hz. The cutoff was selected with reference to previous marker-based three-dimensional kinematic studies using comparable procedures and by visual inspection of raw and filtered trajectories. The filtering procedure was intended to reduce high-frequency marker noise while retaining the overall pattern of the batting movement. Because a formal residual analysis was not performed, the possibility that a fixed 6-Hz cutoff attenuated some higher-frequency components of this explosive task cannot be excluded. Future work should evaluate task-specific cutoff frequencies using residual or sensitivity analyses.

### 2.4. Statistical Analysis

This study used a one-way analysis of variance to compare the differences in segmental angular momentum variables among the no vibration group, the vibration group, the 30% 1RM vibration group, and the 50% 1RM vibration group. The level of significance was set at α = 0.05. When an omnibus ANOVA was significant, Tukey HSD was used for post hoc pairwise comparisons. No additional Bonferroni correction or false discovery rate adjustment was applied across the full set of segmental angular momentum variables.

## 3. Results

### 3.1. Angular Momentum During the Backswing Phase

Significant between-group differences were observed in five angular momentum variables during the backswing phase (Table 1 and Figure 4).

For left shank angular momentum about the *Z*-axis, a significant group effect was observed; F(3,28) = 3.87, *p* = 0.023, η^2^ = 0.346. Post hoc analysis indicated that the 30%1RM+WBV group (813.52 ± 447.03 kg·cm^2^/s) exhibited significantly greater angular momentum than the Non-Vib group (187.71 ± 370.11 kg·cm^2^/s; *p* = 0.023).

A significant group effect was also found for right-upper-arm angular momentum about the *Z*-axis; F(3,28) = 3.09, *p* = 0.048, η^2^ = 0.297. The 30%1RM+WBV group (223.42 ± 137.22 kg·cm^2^/s) demonstrated a significantly greater value than the Non-Vib group (27.09 ± 87.88 kg·cm^2^/s; *p* = 0.032).

For right-forearm angular momentum about the *Z*-axis, the between-group difference was significant; F(3,28) = 4.87, *p* = 0.010, η^2^ = 0.399. The 30%1RM+WBV group (207.84 ± 131.49 kg·cm^2^/s) showed significantly greater angular momentum than both the Non-Vib group (23.76 ± 57.93 kg·cm^2^/s; *p* = 0.007) and the Vib group (60.17 ± 75.95 kg·cm^2^/s; *p* = 0.038).

A significant group effect was identified for trunk angular momentum about the *Y*-axis; F(3,28) = 4.00, *p* = 0.021, η^2^ = 0.353. The 30%1RM+WBV group (788.41 ± 569.23 kg·cm^2^/s) exhibited significantly greater angular momentum than the Non-Vib group (31.51 ± 487.22 kg·cm^2^/s; *p* = 0.043), Vib group (27.95 ± 561.82 kg·cm^2^/s; *p* = 0.042), and 50%1RM+WBV group (−3.17 ± 121.28 kg·cm^2^/s; *p* = 0.041).

For left-forearm angular momentum about the *Y*-axis, a significant group effect was also detected; F(3,28) = 4.02, *p* = 0.020, η^2^ = 0.354. The 30%1RM+WBV group (−113.31 ± 87.94 kg·cm^2^/s) showed a significantly lower value than the Non-Vib group (−7.74 ± 36.76 kg·cm^2^/s; *p* = 0.018); the comparison with the Vib group also reached the statistical threshold (*p* = 0.050). No significant between-group differences were observed for the remaining angular momentum variables during the backswing phase (all *p* > 0.05).

### 3.2. Angular Momentum During the Hip Rotation Initiation Phase

During the hip rotation initiation phase, significant between-group differences were observed in two *Z*-axis angular momentum variables (Table 2 and Figure 5).

A significant group effect was found for trunk angular momentum about the *Z*-axis; F(3,28) = 3.46, *p* = 0.034, η^2^ = 0.320. Post hoc analysis showed that the 30%1RM+WBV group (4396.23 ± 2256.39 kg·cm^2^/s) exhibited significantly greater angular momentum than the 50%1RM+WBV group (1291.03 ± 1099.47 kg·cm^2^/s; *p* = 0.034).

Similarly, left-thigh angular momentum about the *Z*-axis differed significantly among groups; F(3,28) = 3.80, *p* = 0.025, η^2^ = 0.341. The 30%1RM+WBV group (2562.77 ± 732.18 kg·cm^2^/s) demonstrated significantly greater angular momentum than the Vib group (1510.10 ± 537.59 kg·cm^2^/s; *p* = 0.026). No significant between-group differences were found for the other angular momentum variables during this phase (all *p* > 0.05).

### 3.3. Angular Momentum During the Bat–Ball Contact Phase

No significant between-group differences were detected in any of the segmental or whole-body angular momentum variables during the bat–ball contact phase (Table 3; all *p* > 0.05). Although several variables showed numerical differences among the four experimental conditions, none reached statistical significance. Therefore, the intervention-related differences in angular momentum were primarily evident during the earlier backswing and hip rotation initiation phases rather than during the bat–ball contact phase.

### 3.4. Effect Sizes of Significant Angular Momentum Variables

The effect sizes for variables showing significant omnibus ANOVA effects are presented in Figure 6. During the backswing phase, η^2^ values ranged from 0.297 to 0.399, with the largest effect observed for right-forearm angular momentum about the *Z*-axis (η^2^ = 0.399). This was followed by left-forearm angular momentum about the *Y*-axis (η^2^ = 0.354), trunk angular momentum about the *Y*-axis (η^2^ = 0.353), left shank angular momentum about the *Z*-axis (η^2^ = 0.346), and right-upper-arm angular momentum about the *Z*-axis (η^2^ = 0.297).

During the hip rotation initiation phase, effect sizes were η^2^ = 0.341 for left-thigh angular momentum about the *Z*-axis and η^2^ = 0.320 for trunk angular momentum about the *Z*-axis. Collectively, the statistically significant effects were concentrated in the 30%1RM+WBV condition, particularly during the backswing and hip rotation initiation phases.

## 4. Discussion

The present study investigated the acute effects of whole-body vibration (WBV), with and without additional external loading, on segmental angular momentum during different phases of baseball batting. Rather than producing uniform changes throughout the entire swing, the intervention effects were concentrated primarily in the backswing phase and the hip rotation initiation phase. Five angular momentum variables differed significantly among groups during the backswing, whereas two *Z*-axis variables showed significant differences during hip rotation initiation. In contrast, no significant between-group differences were detected during the bat–ball contact phase. These findings suggest that loaded WBV may primarily modify the organization of segmental motion during the preparatory and early acceleration portions of the batting sequence rather than continuously increasing mechanical output throughout the entire swing.

### 4.1. Angular Momentum Adaptations During the Backswing Phase

The backswing represents an important preparatory period in which the batter establishes body position, organizes lower-limb support, and prepares the trunk and upper extremities for subsequent forward acceleration [22,23]. In the present study, this phase demonstrated the greatest number of significant intervention-related differences. Significant group effects were observed for the left shank about the *Z*-axis, right upper arm about the *Z*-axis, right forearm about the *Z*-axis, trunk about the *Y*-axis, and left forearm about the *Y*-axis. Importantly, most of the significant post hoc differences involved the 30%1RM+WBV group, suggesting that moderate external loading combined with vibration produced the most consistent alteration in segmental angular momentum during preparatory loading.

The left-shank *Z*-axis angular momentum was significantly greater in the 30%1RM+WBV group than in the Non-Vib group. Because the lead-side lower limb contributes to body stabilization and preparation for subsequent rotational movement, this result may indicate that moderate-load WBV altered the mechanical contribution of the lower extremity during the preparatory portion of the swing. WBV has previously been proposed to acutely influence neuromuscular responsiveness and the mechanical behavior of the lower extremities [24,25,26]. Nevertheless, angular momentum is determined by both segmental inertia and angular velocity, and therefore the present result should not be interpreted as direct evidence of increased muscular force or motor-unit recruitment. Rather, it reflects a change in the rotational state of the segment before forward swing initiation.

The 30%1RM+WBV condition also resulted in significantly greater right upper-arm *Z*-axis angular momentum than the Non-Vib condition. More notably, right-forearm *Z*-axis angular momentum was significantly greater in the 30%1RM+WBV group than in both the Non-Vib and Vib groups. The right forearm demonstrated the largest effect size among the significant variables (η^2^ = 0.399), indicating that upper-extremity segmental behavior during the preparatory phase was particularly sensitive to the experimental condition. Although WBV was applied primarily through the lower extremities, baseball batting is characterized by coordinated multisegmental motion, and alterations in lower-body preparation may consequently be accompanied by changes in trunk and upper-extremity positioning [27,28,29].

This finding is biomechanically relevant because bat acceleration does not depend on a single joint or segment. Instead, movement is generated through coordinated contributions from the lower extremities, trunk, upper arm, forearm, and ultimately the bat. Therefore, greater forearm angular momentum during the backswing may reflect a different preparatory configuration from which the upper extremity subsequently accelerates. However, whether this altered configuration is mechanically advantageous cannot be determined solely from the present angular momentum measurements.

Another important result involved trunk angular momentum about the *Y*-axis. The 30%1RM+WBV group exhibited significantly greater values than the Non-Vib, Vib, and 50%1RM+WBV groups. The trunk functions as an important mechanical connection between the lower extremities and the upper extremities during batting, and effective trunk motion is generally considered essential for transferring movement from proximal to distal segments [30]. The present finding therefore suggests that moderate-load WBV altered trunk behavior during preparation more consistently than either unloaded vibration or the higher-load vibration condition.

Notably, the 50%1RM+WBV condition did not produce a greater trunk response than the 30%1RM+WBV condition. This result differs from the assumption that increasing the conditioning load should progressively enhance subsequent mechanical output. Instead, the present data suggest that an intermediate external load may provide a more favorable acute stimulus for a rapid and highly coordinated movement such as baseball batting. Previous work on acute conditioning has emphasized that performance responses may depend on the balance between potentiating stimuli and fatigue-related effects rather than on loading magnitude alone [27,28,29]. Accordingly, a 30%1RM load may have provided sufficient mechanical and neuromuscular stimulation while preserving the movement characteristics required for the subsequent high-velocity task.

The left-forearm *Y*-axis angular momentum also differed significantly among groups. The 30%1RM+WBV group demonstrated a more negative value than the Non-Vib group, with the comparison against the Vib group also reaching the statistical threshold. Because angular momentum contains directional information, a more negative value should not be interpreted simply as reduced performance. Based on the laboratory coordinate system, the result instead indicates a greater angular momentum component in the negative Y direction. Consequently, the combined increases and directional changes observed in different segments may reflect a redistribution of angular momentum across the kinetic chain, rather than a generalized increase in all segmental outputs.

Taken together, the backswing results indicate that the acute effect of loaded WBV was expressed as a coordinated modification of lower-limb, trunk, and upper-extremity angular momentum. This pattern is more consistent with altered movement organization than with a simple enhancement of isolated segmental output.

### 4.2. Angular Momentum During Hip Rotation Initiation

Hip rotation initiation represents the transition from preparatory loading to rapid forward acceleration and is a key period during which mechanical output begins to progress from the lower extremities and pelvis toward the trunk and upper extremities [30]. In the present study, significant group effects during this phase were observed primarily in proximal segmental angular momentum.

The 30%1RM+WBV group demonstrated significantly greater trunk *Z*-axis angular momentum than the 50%1RM+WBV group. Because the *Z*-axis represents the vertical axis in the present coordinate system, this variable reflects axial rotational behavior of the trunk during the transition into the forward swing. The greater value observed under the moderate-load condition suggests that combining WBV with 30%1RM may be more compatible with rapid trunk rotation than combining WBV with the heavier 50%1RM load.

Similarly, left-thigh *Z*-axis angular momentum was significantly greater in the 30%1RM+WBV group than in the Vib group. However, the 30%1RM+WBV group did not significantly exceed the Non-Vib group for this variable; therefore, this finding should be interpreted specifically as a difference from the Vib condition rather than as a generalized superiority over all comparison groups. The lead thigh contributes to the establishment of lower-extremity support and rotational organization during the forward swing. Changes in its angular momentum may therefore influence the mechanical conditions under which pelvic and trunk rotation develop. Previous descriptions of the baseball kinetic chain emphasize the importance of coordinated proximal segment motion for subsequent upper-extremity and bat acceleration [31,32].

These results again suggest that the response to WBV was load-dependent rather than linearly load-dependent. If external loading alone determined the magnitude of the acute response, the 50%1RM+WBV condition would be expected to produce the largest values. This was not observed. Instead, the 30%1RM+WBV condition was more consistently associated with greater proximal segmental angular momentum.

One possible explanation is that moderate loading generated sufficient conditioning stimulus without producing the degree of acute mechanical demand associated with the heavier load. From this perspective, the observed pattern may represent the interaction between acute facilitation and fatigue. However, because the present study did not directly quantify muscle activation, central neural drive, muscle contractile properties, or fatigue, this mechanism cannot be confirmed. Accordingly, the findings should be interpreted conservatively as evidence that different WBV loading levels produce distinct biomechanical responses during hip rotation initiation, rather than as direct evidence of enhanced neural activation.

The effect sizes further support the biomechanical relevance of these responses. Left-thigh *Z*-axis angular momentum demonstrated an η^2^ of 0.341, while trunk *Z*-axis angular momentum showed an η^2^ of 0.320. These relatively large effect sizes indicate that the experimental conditions accounted for a meaningful proportion of the variability in proximal rotational behavior, although the small sample size should be considered when interpreting the magnitude of these estimates.

### 4.3. Lack of Significant Differences During the Bat–Ball Contact Phase

In contrast to the backswing and hip rotation initiation phases, no significant differences in segmental or whole-body angular momentum were observed among the four groups during the bat–ball contact phase. This finding is important because it indicates that the influence of WBV was not maintained uniformly through the entire batting sequence.

Bat–ball contact represents the final outcome of a highly coordinated proximal-to-distal movement in which segmental momentum generated earlier in the swing may be transferred, redistributed, or compensated for by other segments before impact [33,34]. Therefore, differences that emerge during preparatory loading or early rotation do not necessarily need to remain visible as differences in individual segment angular momentum at contact.

This may also reflect the task constraints of hitting a stationary ball from a standardized batting tee. Regardless of the preceding movement strategy, participants ultimately had to organize the upper extremities and bat into an appropriate position to contact the ball successfully. Skilled athletes may therefore employ different combinations of segmental motion during earlier phases while converging toward functionally similar mechanical conditions near impact.

Previous biomechanical studies have highlighted the importance of proximal-to-distal sequencing during striking tasks [35]. Within this framework, the absence of significant differences at contact does not necessarily indicate that the earlier intervention-related changes were irrelevant. Instead, they may have influenced the route by which the athlete reached the contact position, while segmental momentum was subsequently redistributed as the movement progressed.

However, the present findings do not support describing WBV as producing a generalized enhancement of angular momentum across the entire batting motion. The acute response was clearly phase-specific, with the greatest effects occurring before the terminal contact phase.

### 4.4. Effect-Size Pattern and the Predominance of the 30%1RM+WBV Condition

An important feature of the present results was the magnitude of the observed effect sizes. Variables showing significant omnibus group effects generally demonstrated η^2^ values of approximately 0.30–0.40. The largest effect was identified for right-forearm *Z*-axis angular momentum during the backswing (η^2^ = 0.399), followed by left-forearm *Y*-axis angular momentum (η^2^ = 0.354), trunk *Y*-axis angular momentum (η^2^ = 0.353), left-shank *Z*-axis angular momentum (η^2^ = 0.346), and left-thigh *Z*-axis angular momentum during hip rotation initiation (η^2^ = 0.341).

The distribution of these effects across lower-limb, trunk, and upper-extremity segments suggests that the acute response was not restricted to the body region directly exposed to vibration. Instead, WBV combined with moderate loading may have modified the organization of the multisegmental batting movement.

Importantly, the 30%1RM+WBV condition accounted for most of the significant pairwise contrasts. This pattern suggests that moderate loading may represent a more favorable acute conditioning stimulus than either vibration alone or vibration combined with a heavier 50%1RM load. Nevertheless, the present findings do not establish an optimal dose–response relationship, and a wider range of loading conditions would be required before an optimal WBV loading prescription could be determined.

### 4.5. Acute Effects and Practical Implications

The timing of the post-intervention assessment is relevant when interpreting the present results. Acute responses to WBV and other conditioning activities are generally considered highly time-dependent, with the balance between facilitation and fatigue potentially changing rapidly during the recovery period [36,37,38].

The present findings may also be considered within the broader theoretical framework of post-activation potentiation (PAP) and post-activation performance enhancement (PAPE) [27,28,36,37,38]. PAP traditionally refers to an acute enhancement of muscle contractile response following a conditioning contraction, whereas PAPE refers more broadly to subsequent improvements in voluntary performance following a conditioning activity. The balance between potentiation-related mechanisms and fatigue has been proposed to influence the magnitude and timing of the subsequent performance response. In the present study, however, electrically evoked twitch responses, muscle activation, and contractile properties were not measured; therefore, the observed biomechanical changes cannot be interpreted as direct evidence of PAP. Similarly, because the primary outcomes were segmental angular momentum variables rather than direct improvements in batting performance, the findings should not be considered definitive evidence of PAPE. Rather, the PAP/PAPE framework provides a theoretical context for understanding why moderate and heavier WBV loading conditions may produce different acute biomechanical responses.

In the present study, the batting task was performed immediately after the conditioning protocol, and therefore the observed segmental angular momentum responses represent the immediate biomechanical state following the intervention.

From an applied perspective, the findings suggest that 30%1RM combined with WBV may be considered as a potential pre-activity conditioning strategy when the objective is to modify preparatory and proximal rotational characteristics of batting. In particular, the moderate-load condition was associated with differences in the shank, thigh, trunk, upper arm, and forearm during the backswing and hip rotation initiation phases.

For adolescent athletes, however, WBV protocols should be implemented cautiously. Appropriate supervision, individualized loading, standardized lower-limb posture, and control of vibration exposure are particularly important in developing athletes [39]. The present results therefore support further investigation of moderate-load WBV as a sport-specific warm-up or priming strategy, rather than immediate routine implementation without additional evidence.

It should also be emphasized that changes in angular momentum cannot automatically be equated with improved batting performance. Whether the biomechanical changes observed under the 30%1RM+WBV condition ultimately contribute to greater bat velocity, higher ball exit velocity, improved accuracy, or competitive hitting effectiveness requires direct examination.

### 4.6. Limitations

Several limitations should be considered. First, the number of participants included in the angular momentum analysis was relatively small, which may reduce statistical power and increase uncertainty in the estimated effect sizes. Larger samples are therefore required to confirm the stability of the observed group differences.

Second, only the acute effects of the interventions were investigated. Consequently, the present findings cannot be extrapolated to chronic training adaptations. Repeated exposure to WBV combined with external loading may result in different biomechanical or neuromuscular adaptations.

Third, although segmental angular momentum provides useful information regarding the rotational behavior of different body segments, the underlying physiological mechanisms were not directly assessed. Measures such as surface electromyography, joint moments, ground reaction forces, and muscle-tendon behavior would help clarify whether the observed changes are related to altered muscle activation, joint stiffness, force production, or intersegmental coordination.

Fourth, the trial with the highest ball exit velocity was selected for biomechanical analysis. Although this approach was used to characterize each athlete’s maximal batting performance, a single representative trial may not fully capture within-participant variability.

Fifth, the study involved adolescent male national-level baseball players. The results may therefore not be directly applicable to female athletes, adult professional players, or athletes of different competitive levels.

Finally, multiple segmental angular momentum variables were examined across different axes and phases. Therefore, individual significant findings should be interpreted together with the observed effect sizes and the overall biomechanical pattern rather than solely on the basis of statistical significance. No additional Bonferroni correction or false discovery rate adjustment was applied across the full set of outcome variables; consequently, the possibility of inflated Type I error cannot be excluded. Accordingly, these findings should be considered exploratory and should be confirmed in larger independent samples.

### 4.7. Summary

The present study demonstrates that the acute biomechanical response to WBV combined with external loading is phase-specific and segment-dependent. Intervention-related differences in angular momentum were concentrated during the backswing and hip rotation initiation phases, whereas no significant group differences were observed during bat–ball contact.

Among the tested conditions, the 30%1RM+WBV protocol produced the most consistent segmental responses, particularly in the lower extremity, trunk, and upper extremity during preparatory loading and early rotational acceleration. Increasing the load to 50%1RM did not produce systematically greater responses, suggesting that the acute biomechanical effect of loaded WBV does not follow a simple load-dependent pattern.

Overall, moderate-load WBV appears to modify the distribution and directional organization of angular momentum within the batting kinetic chain. Further research incorporating larger samples, neuromuscular measurements, kinetic variables, and longitudinal training protocols is needed to determine whether these acute changes translate into meaningful improvements in baseball batting performance.

## 5. Conclusions

This study demonstrates that the acute biomechanical effects of whole-body vibration combined with external loading are phase-specific and segment-dependent during baseball batting. Significant differences in segmental angular momentum were primarily observed during the backswing and hip rotation initiation phases, whereas no significant between-group differences were detected during the bat–ball contact phase. These findings indicate that the interventions mainly modified the organization of segmental motion during the preparatory and early rotational portions of the swing rather than producing a uniform increase in mechanical output throughout the entire batting sequence.

Among the tested conditions, 30%1RM combined with WBV produced the most consistent biomechanical responses. During the backswing phase, this condition was associated with significant differences in the angular momentum of the left shank, trunk, right upper arm, right forearm, and left forearm. During hip rotation initiation, the 30%1RM+WBV condition was also associated with greater trunk and left-thigh *Z*-axis angular momentum relative to selected comparison groups. In contrast, increasing the external load to 50%1RM did not result in systematically greater segmental responses. Therefore, the acute influence of loaded WBV does not appear to follow a simple linear load–response relationship.

The present findings suggest that moderate external loading combined with WBV may alter the distribution, direction, and coordination of angular momentum across multiple body segments, particularly during the phases preceding bat–ball contact. However, these changes should not be interpreted as direct evidence of increased muscular force, improved energy-transfer efficiency, or enhanced batting performance, because the physiological mechanisms underlying the observed responses were not directly assessed.

From a practical perspective, 30%1RM+WBV may warrant further investigation as a short-duration pre-activity conditioning strategy for baseball athletes. Nevertheless, its application should remain individualized and conservative, particularly in adolescent players. Further studies should examine larger samples, additional loading levels, different post-intervention recovery intervals, and direct measures of neuromuscular activation, kinetics, bat velocity, and batted-ball exit velocity to determine whether the acute biomechanical changes observed in the present study translate into meaningful improvements in batting performance.

## Figures and Tables

**Figure 1:**
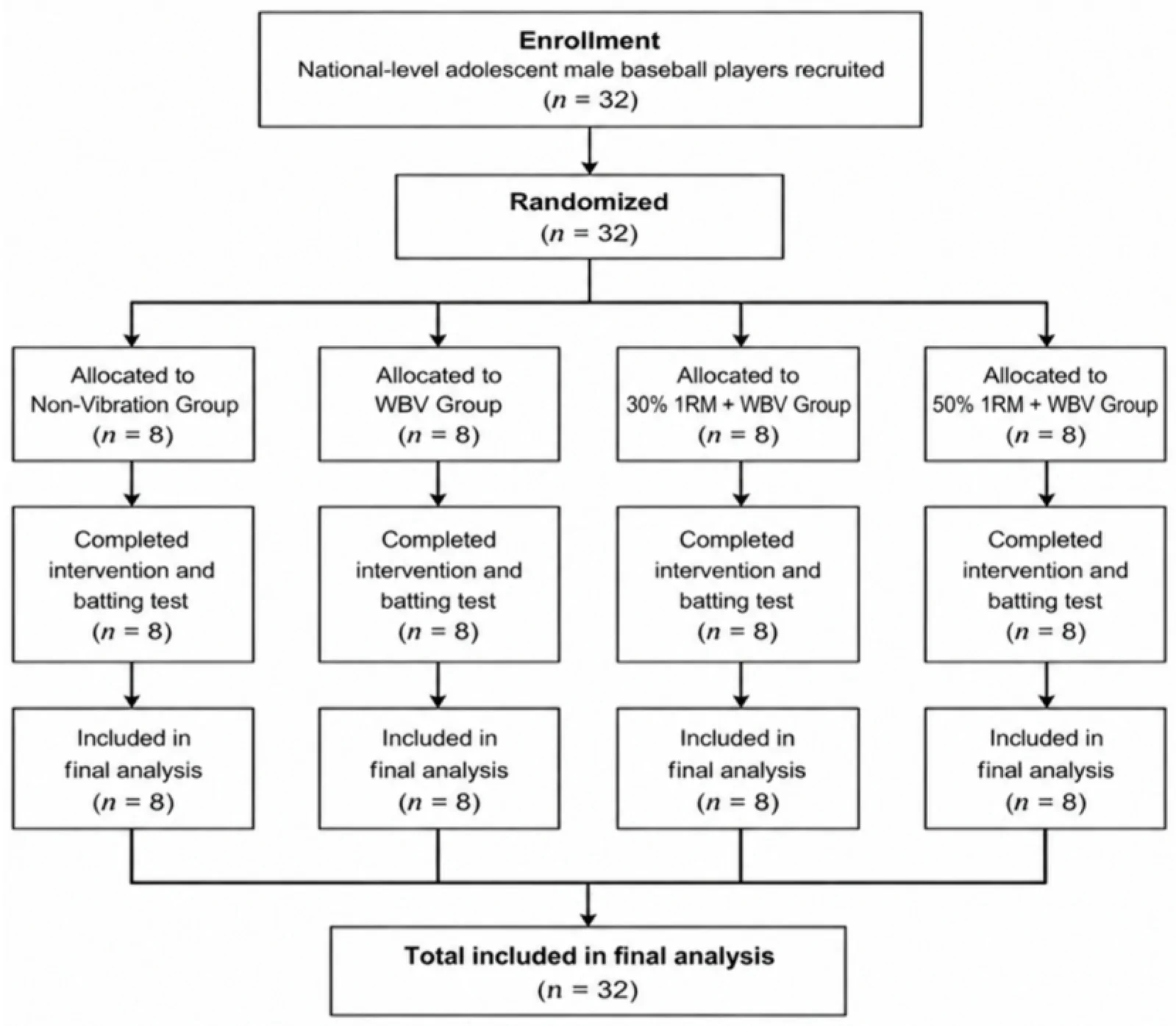
CONSORT-style flow diagram of participant recruitment, randomization, intervention allocation, follow-up, and final analysis.

**Figure 2:**
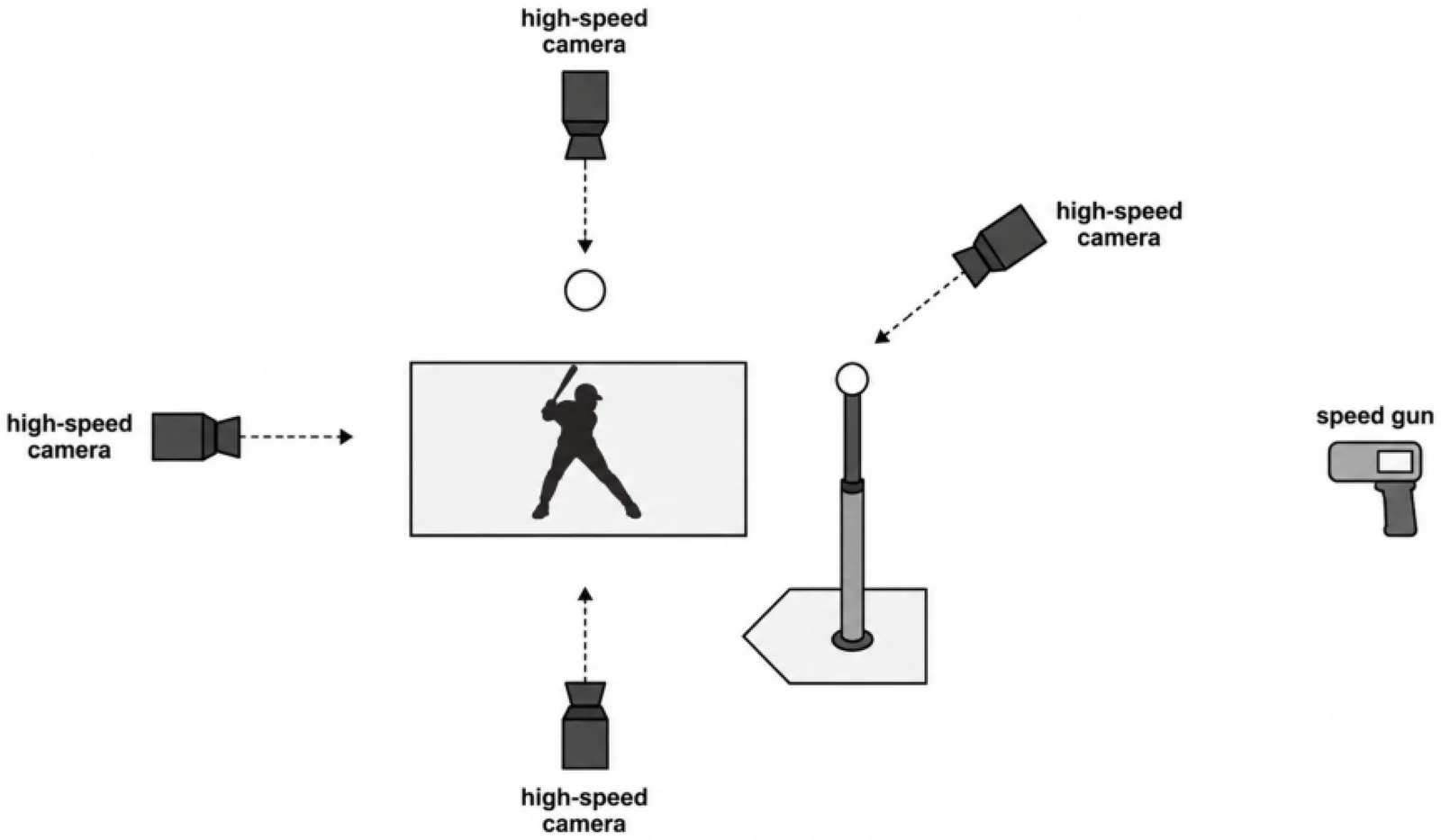
Experimental setup for three-dimensional baseball batting analysis. The high-speed camera system surrounded the calibrated hitting area, while the radar speed gun was aligned with the projected batted-ball trajectory. This figure was originally designed by the authors with the assistance of ChatGPT-6 Astra for visual generation. The content and biomechanical concepts were specified, reviewed, and approved by the authors.

**Figure 3:**
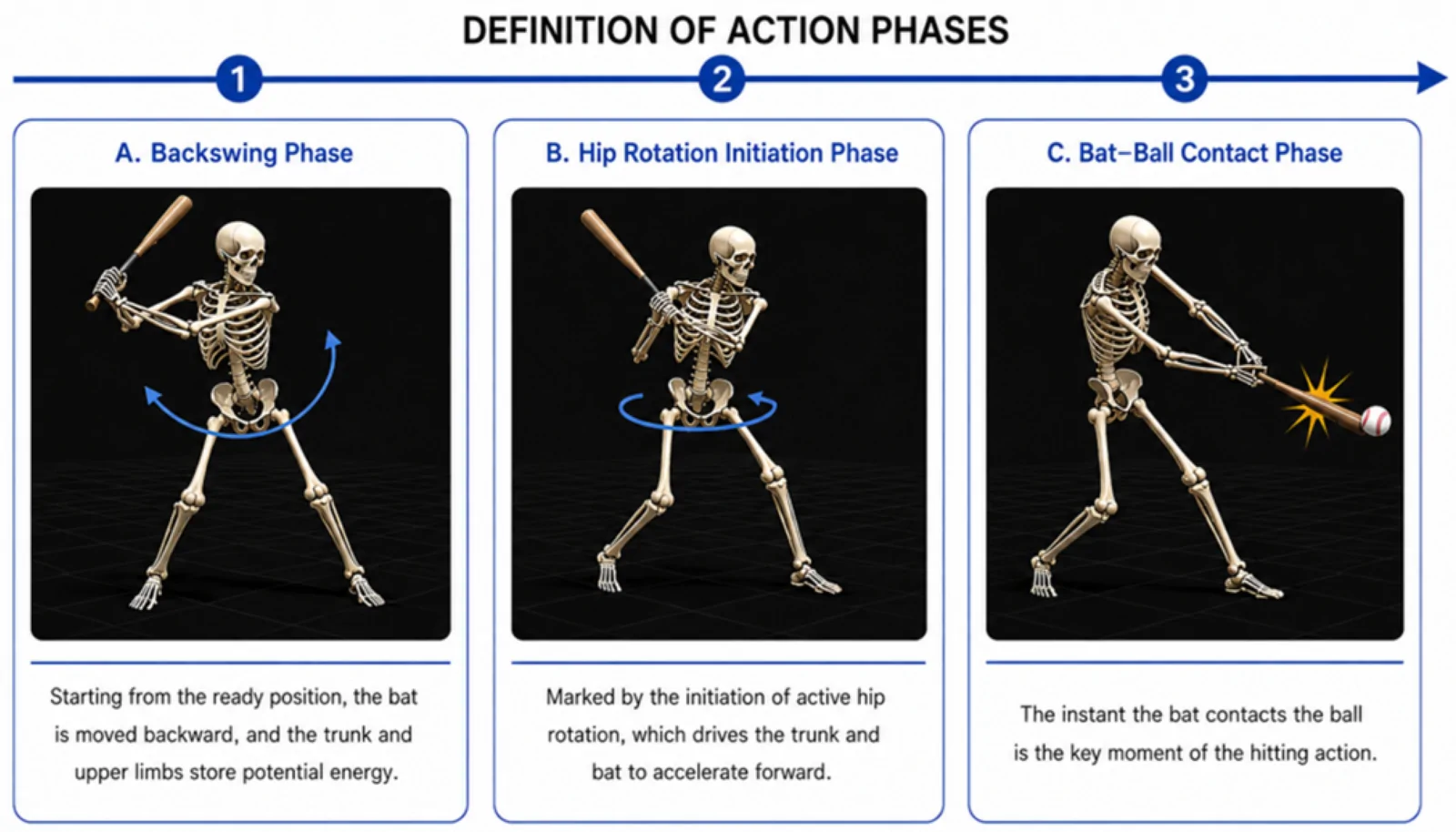
Schematic representation of the three batting phases used for biomechanical analysis: (**A**) Backswing Phase, (**B**) Hip Rotation Initiation Phase, and (**C**) Bat–Ball Contact Phase. This figure was originally designed by the authors with the assistance of ChatGPT-6 Astra for visual generation. The content and biomechanical concepts were specified, reviewed, and approved by the authors.

**Figure 4:**
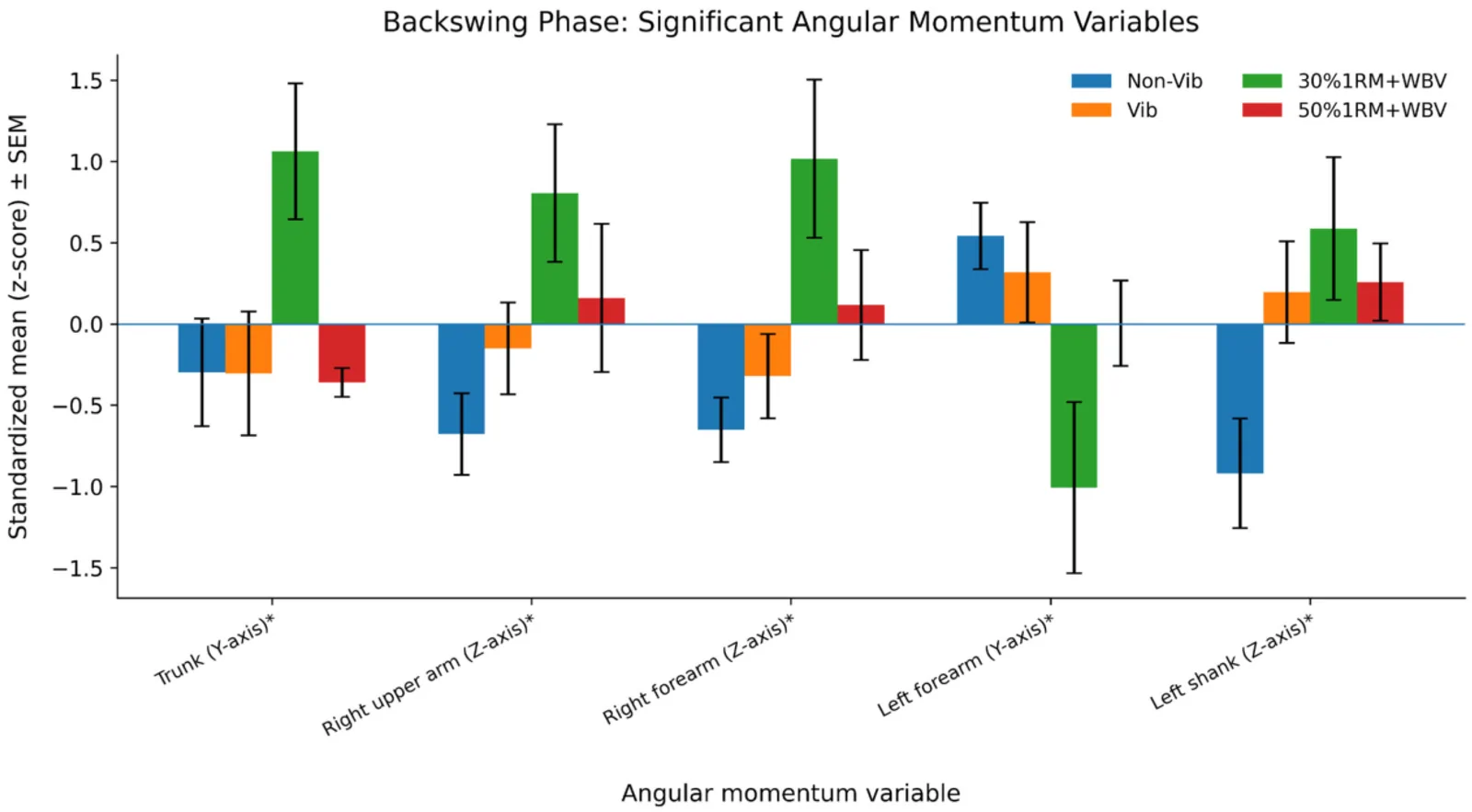
Standardized angular momentum variables showing significant between-group differences during the backswing phase. Values are presented as standardized means (z-scores) ± SEM. * indicates a significant omnibus group effect (*p* < 0.05).

**Figure 5:**
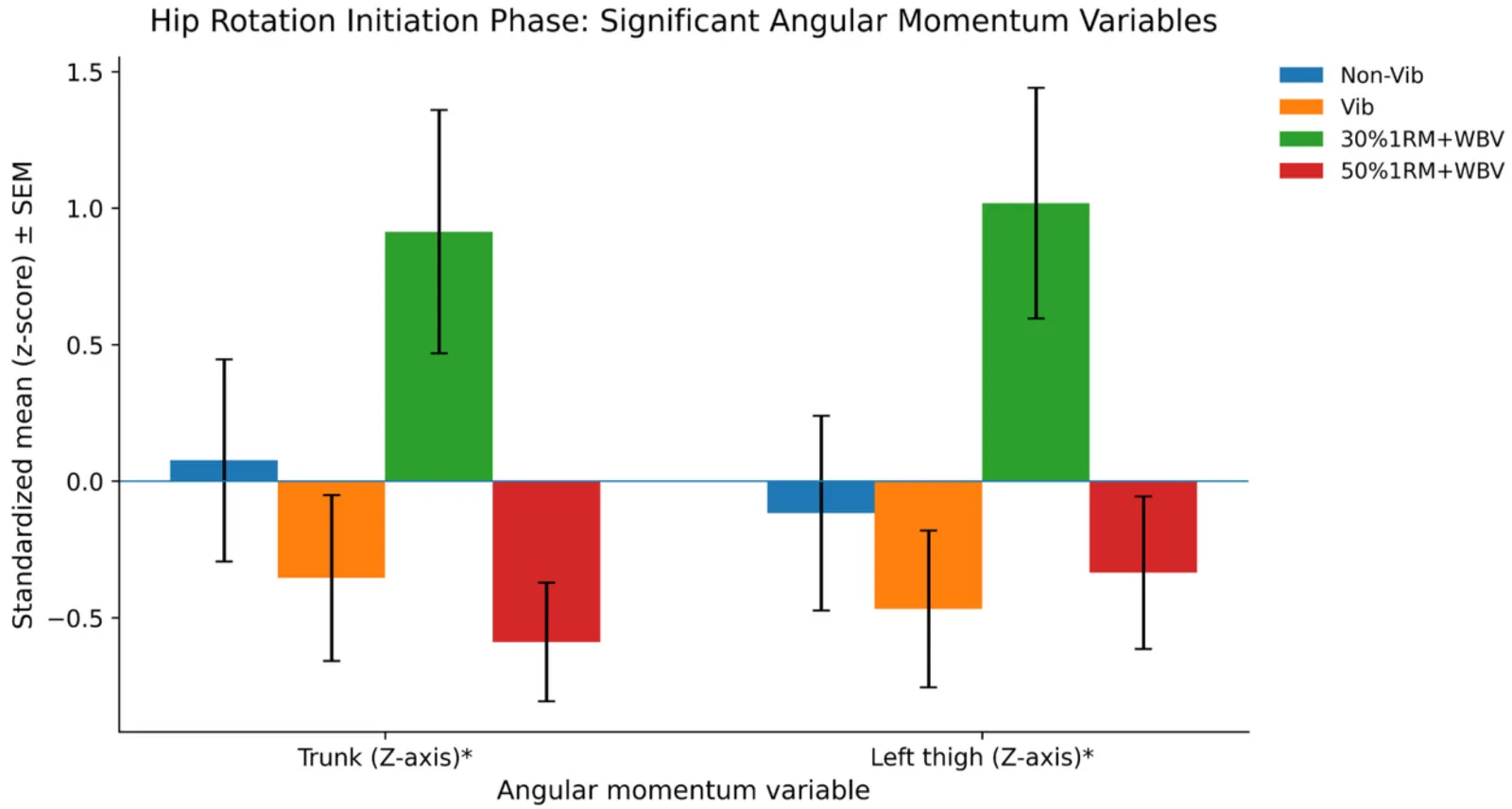
Standardized angular momentum variables with significant between-group differences during the hip rotation initiation phase. Values are presented as standardized means (z-scores) ± SEM. * indicates a significant omnibus group effect (*p* < 0.05).

**Figure 6:**
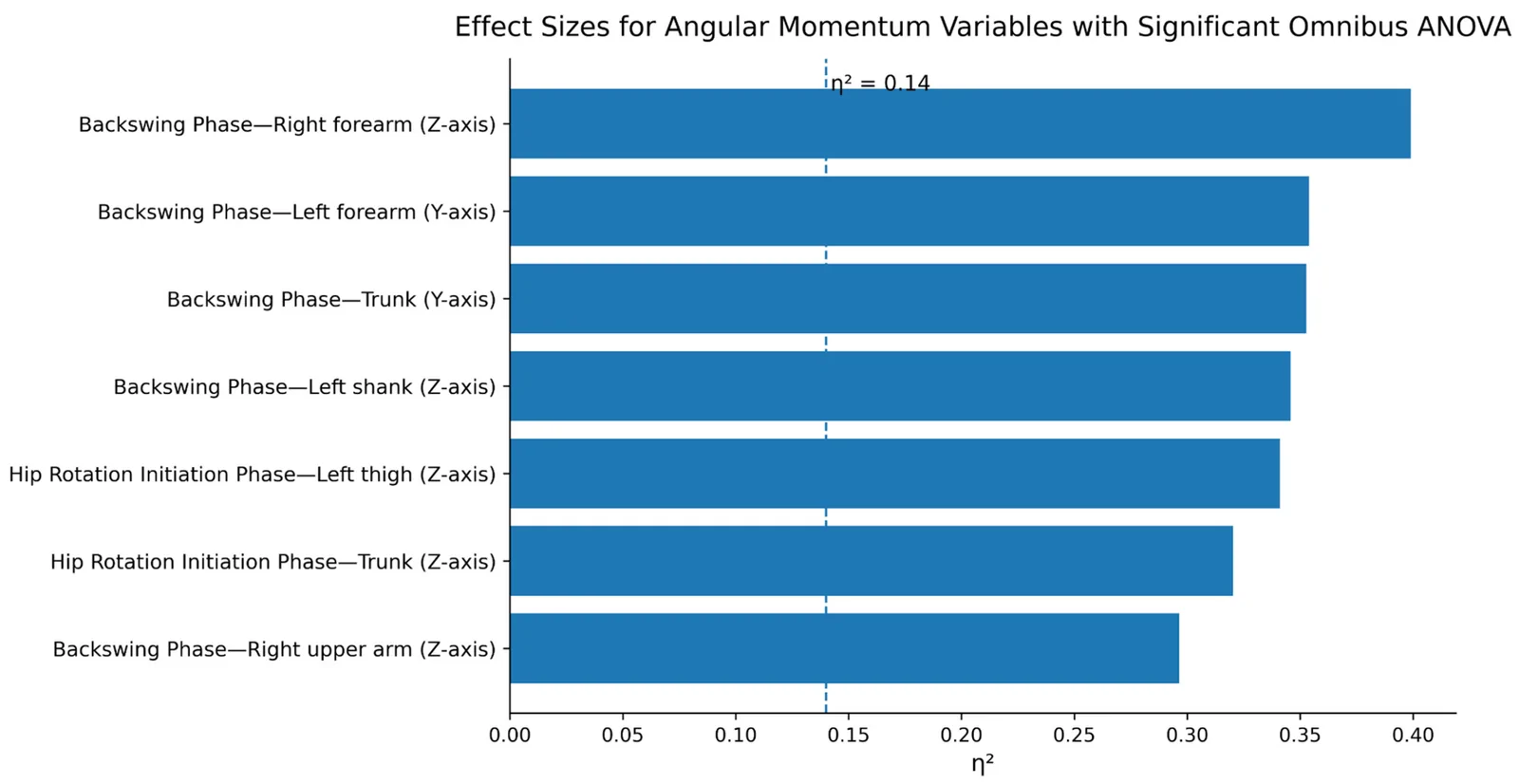
Effect sizes (η^2^) for angular momentum variables demonstrating significant omnibus ANOVA effects. The dashed vertical line at η^2^ = 0.14 indicates the conventional reference value for a large effect.

**Table 1:** Angular Momentum During the Backswing Phase (kg·cm^2^/s).

Variable	Non-Vib (*n* = 8) Mean ± SD	Vib (*n* = 8) Mean ± SD	30% 1RM + WBV (*n* = 8) Mean ± SD	50% 1RM + WBV (*n* = 8) Mean ± SD	F(3,28)	*p*	η^2^	Post Hoc Comparison
Trunk (*Z*-axis)	267.78 ± 866.00	653.56 ± 978.41	336.52 ± 899.29	810.28 ± 926.08	0.5	0.684	0.064	
Right thigh (*Z*-axis)	41.69 ± 236.40	40.02 ± 271.69	113.77 ± 166.35	114.67 ± 270.55	0.2	0.896	0.026	
Right shank (*Z*-axis)	29.01 ± 53.12	−10.05 ± 60.58	38.35 ± 68.54	56.52 ± 38.66	1.64	0.209	0.183	
Left thigh (*Z*-axis)	251.85 ± 457.16	764.47 ± 490.58	841.82 ± 617.78	909.95 ± 238.78	2.71	0.070	0.27	
Left shank (*Z*-axis)	187.71 ± 370.11	650.90 ± 344.18	813.52 ± 447.03	676.50 ± 242.11	3.87	0.023 *	0.346	3 > 1 (*p* = 0.023)
Right upper arm (*Z*-axis)	27.09 ± 87.88	96.81 ± 98.95	223.42 ± 137.22	137.96 ± 147.80	3.09	0.048 *	0.297	3 > 1 (*p* = 0.032)
Right forearm (*Z*-axis)	23.76 ± 57.93	60.17 ± 75.95	207.84 ± 131.49	108.52 ± 91.44	4.87	0.010 **	0.399	3 > 1 (*p* = 0.007); 3 > 2 (*p* = 0.038)
Left upper arm (*Z*-axis)	28.66 ± 43.71	51.36 ± 70.62	−41.34 ± 88.67	65.17 ± 86.21	2.55	0.082	0.258	
Left forearm (*Z*-axis)	26.07 ± 27.04	41.58 ± 59.67	36.19 ± 48.48	61.05 ± 26.74	0.73	0.543	0.091	
Whole body (*Z*-axis)	937.31 ± 1636.14	2447.90 ± 1696.09	2973.61 ± 1829.12	3150.73 ± 1622.59	2.34	0.101	0.242	
Trunk (*Y*-axis)	31.51 ± 487.22	27.95 ± 561.82	788.41 ± 569.23	−3.17 ± 121.28	4	0.021 *	0.353	3 > 1 (*p* = 0.043); 3 > 2 (*p* = 0.042); 3 > 4 (*p* = 0.041)
Right thigh (*Y*-axis)	13.21 ± 114.22	30.46 ± 139.12	61.01 ± 137.72	39.58 ± 100.02	0.17	0.918	0.022	
Right shank (*Y*-axis)	−2.87 ± 16.93	4.95 ± 12.47	1.66 ± 20.89	−2.49 ± 19.65	0.3	0.823	0.04	
Left thigh (*Y*-axis)	10.76 ± 134.58	−144.13 ± 265.65	−7.51 ± 266.99	−205.94 ± 160.54	1.53	0.234	0.173	
Left shank (*Y*-axis)	13.92 ± 44.83	−50.80 ± 156.28	0.72 ± 109.82	−36.36 ± 85.19	0.54	0.660	0.069	
Right upper arm (*Y*-axis)	−23.08 ± 96.69	−72.16 ± 117.15	−156.07 ± 204.01	−69.08 ± 126.71	1	0.410	0.12	
Right forearm (*Y*-axis)	−34.25 ± 75.83	−57.79 ± 126.92	−224.67 ± 205.92	−75.09 ± 99.08	2.59	0.078	0.261	
Left upper arm (*Y*-axis)	4.86 ± 32.86	−0.09 ± 44.56	23.66 ± 24.89	−17.57 ± 86.47	0.65	0.594	0.081	
Left forearm (*Y*-axis)	−7.74 ± 36.76	−22.98 ± 55.77	−113.31 ± 87.94	−44.36 ± 43.86	4.02	0.020 *	0.354	1 > 3 (*p* = 0.018); 2 > 3 (*p* = 0.050)
Whole body (*Y*-axis)	48.84 ± 928.53	−205.51 ± 760.22	306.92 ± 1269.52	−477.15 ± 478.09	0.85	0.481	0.104	
Trunk (*X*-axis)	−909.53 ± 1405.42	−1848.01 ± 872.23	−2378.98 ± 1794.60	−1785.34 ± 1475.67	1.24	0.319	0.145	
Right thigh (*X*-axis)	−111.54 ± 205.63	−201.71 ± 123.31	−254.35 ± 227.53	−252.67 ± 132.80	0.94	0.436	0.114	
Right shank (*X*-axis)	−12.76 ± 30.10	−8.29 ± 20.34	−21.52 ± 28.41	−33.64 ± 45.74	0.78	0.517	0.096	
Left thigh (*X*-axis)	−297.97 ± 290.85	−543.15 ± 406.52	−678.74 ± 413.41	−428.69 ± 404.20	1.19	0.338	0.139	
Left shank (*X*-axis)	−154.60 ± 188.12	−243.47 ± 274.10	−397.41 ± 242.08	−285.83 ± 314.44	0.99	0.415	0.119	
Right upper arm (*X*-axis)	−82.10 ± 78.12	−134.70 ± 96.37	−208.17 ± 213.61	−235.74 ± 92.44	1.92	0.155	0.208	
Right forearm (*X*-axis)	−23.78 ± 26.91	−55.71 ± 76.94	−61.54 ± 151.33	−131.38 ± 83.92	1.51	0.240	0.171	
Left upper arm (*X*-axis)	−69.62 ± 76.60	−107.44 ± 70.01	16.24 ± 185.86	−81.59 ± 100.23	1.37	0.277	0.158	
Left forearm (*X*-axis)	−38.16 ± 60.51	−42.70 ± 92.59	115.16 ± 193.62	−27.03 ± 43.44	2.9	0.058	0.283	
Whole body (*X*-axis)	−1913.62 ± 2101.51	−3548.50 ± 1778.31	−4297.38 ± 2959.38	−3637.58 ± 2076.10	1.36	0.281	0.156	

Note. Data are presented as Mean ± SD. * *p* < 0.05; ** *p* < 0.01. Axis conventions: *X*-axis, mediolateral (left–right); *Y*-axis, anteroposterior (forward–backward); *Z*-axis, vertical (up–down). Post hoc comparisons were performed using Tukey HSD.

**Table 2:** Angular Momentum During the Hip Rotation Initiation Phase (kg·cm^2^/s).

Variable	Non-Vib (*n* = 8) Mean ± SD	Vib (*n* = 8) Mean ± SD	30% 1RM + WBV (*n* = 8) Mean ± SD	50% 1RM + WBV (*n* = 8) Mean ± SD	F(3,28)	*p*	η^2^	Post Hoc Comparison
Trunk (*Z*-axis)	2664.81 ± 2023.29	1774.84 ± 1660.54	4396.23 ± 2256.39	1291.03 ± 1099.47	3.46	0.034 *	0.32	3 > 4 (*p* = 0.034)
Right thigh (*Z*-axis)	−71.51 ± 644.17	−188.60 ± 267.22	186.11 ± 472.35	−384.09 ± 207.24	1.78	0.181	0.195	
Right shank (*Z*-axis)	56.15 ± 250.49	36.12 ± 107.93	192.11 ± 189.52	−19.35 ± 97.80	1.59	0.220	0.178	
Left thigh (*Z*-axis)	1758.43 ± 668.10	1510.10 ± 537.59	2562.77 ± 732.18	1604.10 ± 483.89	3.8	0.025 *	0.341	3 > 2 (*p* = 0.026)
Left shank (*Z*-axis)	379.51 ± 248.71	520.44 ± 269.93	730.45 ± 410.21	822.77 ± 597.76	1.67	0.202	0.186	
Right upper arm (*Z*-axis)	−240.79 ± 167.05	−188.66 ± 289.95	−289.74 ± 289.02	−166.59 ± 257.95	0.29	0.832	0.038	
Right forearm (*Z*-axis)	−195.62 ± 190.88	−146.95 ± 216.37	−238.67 ± 283.56	−164.49 ± 215.93	0.2	0.896	0.026	
Left upper arm (*Z*-axis)	373.58 ± 324.06	238.56 ± 193.30	490.55 ± 479.71	235.00 ± 312.36	0.83	0.494	0.101	
Left forearm (*Z*-axis)	97.47 ± 148.79	69.63 ± 140.47	95.69 ± 327.32	48.73 ± 208.47	0.07	0.973	0.01	
Whole body (*Z*-axis)	5281.09 ± 3784.76	4410.50 ± 2601.46	9237.60 ± 4922.90	4032.16 ± 2963.92	2.63	0.075	0.264	
Trunk (*Y*-axis)	−1115.09 ± 1814.72	348.18 ± 981.18	−1029.69 ± 1723.44	635.52 ± 466.77	2.84	0.062	0.279	
Right thigh (*Y*-axis)	300.00 ± 341.43	386.35 ± 145.94	339.84 ± 94.88	489.59 ± 258.00	0.77	0.523	0.095	
Right shank (*Y*-axis)	66.64 ± 83.53	67.70 ± 36.87	66.69 ± 44.82	96.99 ± 74.81	0.35	0.793	0.045	
Left thigh (*Y*-axis)	−624.31 ± 309.86	−208.98 ± 304.24	−724.24 ± 609.31	−221.51 ± 159.11	3.26	0.051	0.307	
Left shank (*Y*-axis)	−46.57 ± 46.44	85.85 ± 164.63	−2.37 ± 166.04	46.96 ± 131.23	1.26	0.312	0.147	
Right upper arm (*Y*-axis)	282.65 ± 466.64	382.56 ± 240.07	666.30 ± 274.49	354.24 ± 216.44	1.72	0.192	0.19	
Right forearm (*Y*-axis)	259.80 ± 419.10	292.71 ± 256.57	586.07 ± 327.98	329.45 ± 164.35	1.43	0.260	0.163	
Left upper arm (*Y*-axis)	−79.69 ± 282.97	28.15 ± 97.62	−39.28 ± 261.13	47.03 ± 205.83	0.46	0.712	0.059	
Left forearm (*Y*-axis)	110.71 ± 226.36	124.41 ± 171.45	278.13 ± 144.75	170.48 ± 127.31	1.19	0.335	0.14	
Whole body (*Y*-axis)	−955.80 ± 3727.44	1453.19 ± 1476.92	175.59 ± 2415.04	1997.14 ± 859.75	1.97	0.147	0.212	
Trunk (*X*-axis)	−4395.43 ± 3993.83	−5324.07 ± 1300.65	−5623.01 ± 1547.23	−5091.17 ± 1175.05	0.32	0.810	0.042	
Right thigh (*X*-axis)	−1018.71 ± 469.95	−752.32 ± 146.12	−942.69 ± 408.43	−815.45 ± 476.65	0.64	0.599	0.08	
Right shank (*X*-axis)	−233.01 ± 128.35	−174.14 ± 68.28	−211.24 ± 74.18	−213.11 ± 139.92	0.37	0.778	0.048	
Left thigh (*X*-axis)	−414.08 ± 762.95	−1151.44 ± 835.91	−784.60 ± 789.60	−1052.95 ± 591.23	1.31	0.297	0.151	
Left shank (*X*-axis)	−89.45 ± 186.79	−621.07 ± 742.00	−323.29 ± 511.48	−642.45 ± 562.10	1.6	0.217	0.179	
Right upper arm (*X*-axis)	−608.20 ± 441.66	−756.41 ± 163.70	−879.12 ± 239.58	−591.37 ± 218.30	1.36	0.282	0.156	
Right forearm (*X*-axis)	−452.20 ± 323.82	−487.73 ± 135.12	−646.83 ± 247.24	−379.81 ± 203.16	1.35	0.284	0.156	
Left upper arm (*X*-axis)	−574.45 ± 484.49	−526.07 ± 201.02	−698.42 ± 199.38	−493.11 ± 231.57	0.51	0.678	0.065	
Left forearm (*X*-axis)	−403.56 ± 349.79	−357.02 ± 190.59	−543.15 ± 280.40	−339.34 ± 164.14	0.77	0.521	0.095	
Whole body (*X*-axis)	−9489.77 ± 7965.10	−11,995.15 ± 3467.25	−12,801.89 ± 2959.65	−11,335.64 ± 2142.29	0.56	0.646	0.071	

Note. Data are presented as Mean ± SD. * *p* < 0.05. Axis conventions: *X*-axis, mediolateral (left–right); *Y*-axis, anteroposterior (forward–backward); *Z*-axis, vertical (up–down). Post hoc comparisons were performed using Tukey HSD.

**Table 3:** Angular Momentum During the Bat–Ball Contact Phase (kg·cm^2^/s).

Variable	Non-Vib (*n* = 8) Mean ± SD	Vib (*n* = 8) Mean ± SD	30% 1RM + WBV (*n* = 8) Mean ± SD	50% 1RM + WBV (*n* = 8) Mean ± SD	F(3,28)	*p*	η^2^	Post Hoc Comparison
Trunk (*Z*-axis)	−5233.34 ± 2468.77	−5175.26 ± 1881.73	−6722.83 ± 3214.45	−4342.91 ± 1603.86	1.06	0.386	0.126	
Right thigh (*Z*-axis)	560.95 ± 744.76	795.67 ± 704.79	258.29 ± 680.73	884.79 ± 640.49	0.99	0.415	0.119	
Right shank (*Z*-axis)	225.71 ± 487.43	350.95 ± 203.87	129.18 ± 241.30	355.34 ± 637.21	0.41	0.749	0.053	
Left thigh (*Z*-axis)	−1123.56 ± 413.66	−1303.67 ± 937.50	−2171.45 ± 1442.66	−862.41 ± 238.80	2.52	0.084	0.256	
Left shank (*Z*-axis)	193.92 ± 282.01	−52.27 ± 454.86	−417.00 ± 430.08	−160.83 ± 477.68	2.41	0.094	0.248	
Right upper arm (*Z*-axis)	101.71 ± 143.33	30.05 ± 219.79	158.19 ± 327.47	−4.03 ± 172.64	0.65	0.590	0.082	
Right forearm (*Z*-axis)	878.86 ± 176.00	820.86 ± 223.88	872.74 ± 363.80	840.78 ± 161.78	0.09	0.967	0.012	
Left upper arm (*Z*-axis)	97.29 ± 401.14	323.66 ± 367.29	19.77 ± 419.10	341.73 ± 428.17	1.02	0.403	0.122	
Left forearm (*Z*-axis)	890.27 ± 171.44	1115.59 ± 202.51	1061.23 ± 345.72	1143.58 ± 293.76	1.32	0.292	0.153	
Whole body (*Z*-axis)	−2934.15 ± 3971.23	−2775.53 ± 3287.87	−6743.12 ± 4794.35	−1602.55 ± 2375.99	2.2	0.117	0.23	
Trunk (*Y*-axis)	2728.19 ± 2418.56	1026.28 ± 1086.09	1525.27 ± 2516.94	493.81 ± 492.48	1.78	0.181	0.195	
Right thigh (*Y*-axis)	−441.17 ± 447.13	−549.45 ± 325.00	−422.11 ± 252.48	−563.92 ± 327.90	0.28	0.842	0.036	
Right shank (*Y*-axis)	−108.36 ± 130.17	−132.38 ± 62.21	−109.93 ± 72.70	−131.85 ± 94.75	0.13	0.942	0.017	
Left thigh (*Y*-axis)	365.55 ± 298.00	202.33 ± 337.56	539.26 ± 654.94	−0.92 ± 43.22	2.11	0.128	0.223	
Left shank (*Y*-axis)	−5.03 ± 91.32	−44.01 ± 101.79	9.75 ± 83.51	−61.18 ± 88.40	0.8	0.505	0.099	
Right upper arm (*Y*-axis)	−182.80 ± 344.66	−268.58 ± 208.18	−554.68 ± 353.73	−200.34 ± 146.92	2.37	0.098	0.244	
Right forearm (*Y*-axis)	−610.34 ± 325.74	−599.41 ± 238.19	−767.39 ± 383.12	−589.16 ± 103.85	0.54	0.658	0.069	
Left upper arm (*Y*-axis)	−312.60 ± 226.07	−565.14 ± 172.54	−429.19 ± 232.00	−615.23 ± 330.62	2.1	0.130	0.222	
Left forearm (*Y*-axis)	−734.10 ± 157.69	−843.61 ± 213.77	−890.80 ± 363.45	−869.13 ± 238.83	0.52	0.673	0.066	
Whole body (*Y*-axis)	499.06 ± 3714.00	−2195.10 ± 1097.94	−1652.90 ± 2865.05	−2886.75 ± 1410.02	2.24	0.112	0.234	
Trunk (*X*-axis)	7447.97 ± 4076.62	9751.94 ± 2805.63	8881.97 ± 2920.93	9034.61 ± 2779.61	0.63	0.606	0.079	
Right thigh (*X*-axis)	1094.03 ± 746.37	831.99 ± 303.70	990.65 ± 917.88	867.75 ± 660.64	0.21	0.889	0.028	
Right shank (*X*-axis)	229.35 ± 248.53	182.10 ± 113.06	192.70 ± 127.35	197.44 ± 126.63	0.1	0.957	0.014	
Left thigh (*X*-axis)	796.96 ± 875.72	1521.25 ± 649.98	1208.50 ± 651.58	1463.49 ± 228.04	1.72	0.192	0.19	
Left shank (*X*-axis)	−15.51 ± 238.38	274.95 ± 381.05	175.29 ± 206.95	387.21 ± 319.32	2.19	0.118	0.23	
Right upper arm (*X*-axis)	684.81 ± 497.81	911.35 ± 238.75	1127.15 ± 233.19	747.07 ± 288.16	2.15	0.123	0.227	
Right forearm (*X*-axis)	357.52 ± 474.56	432.15 ± 168.63	649.92 ± 254.21	387.39 ± 267.34	1.08	0.377	0.129	
Left upper arm (*X*-axis)	1051.36 ± 576.81	1111.68 ± 240.29	1070.32 ± 389.24	1056.40 ± 341.92	0.03	0.993	0.004	
Left forearm (*X*-axis)	304.04 ± 480.01	284.21 ± 236.25	417.11 ± 318.72	260.71 ± 319.03	0.24	0.871	0.031	
Whole body (*X*-axis)	13,169.05 ± 8054.93	17,071.08 ± 4225.35	16,597.47 ± 5242.75	16,107.25 ± 4609.63	0.63	0.602	0.079	

Note. Data are presented as Mean ± SD. Axis conventions: *X*-axis, mediolateral (left–right); *Y*-axis, anteroposterior (forward–backward); *Z*-axis, vertical (up–down). Post hoc comparisons were performed using Tukey HSD.

## Data Availability

The data supporting the findings of this study are available from the corresponding author upon reasonable request.

## References

[1] Welch C.M., Banks S.A., Cook F.F., Draovitch P. (1995). Hitting a baseball: A biomechanical description. J. Orthop. Sports Phys. Ther..

[2] Perrett C.S. (2023). The contribution of lower-body kinematics to pitching and hitting performance in baseball: Utilizing the openbiomechanics project. J. Appl. Biomech..

[3] Nicholls R.L., Elliott B.C., Miller K., Koh M. (2003). Bat kinematics in baseball: Implications for ball exit velocity and player safety. J. Appl. Biomech..

[4] Li F.Y., Guo C.G., Li H.S., Xu H.R., Sun P. (2023). A systematic review and net meta-analysis of the effects of different warm-up methods on the acute effects of lower limb explosive strength. BMC Sports Sci. Med. Rehabil..

[5] Peng Y.C., Guo Y.T., Wu J.C., Hou W.H. (2025). Effects of Whole-Body Vibration on Exercise Performance among Athletes: A Systematic Review and Meta-Analysis of Randomized Controlled Trials. J. Hum. Kinet..

[6] Corum M., Topkara B., Kokce M., Ozkan M., Bucak O.F., Ayture L., Türker K.S. (2022). The reflex mechanism underlying the neuromuscular effects of whole-body vibration: Is it the tonic vibration reflex?. J. Musculoskelet. Neuronal Interact..

[7] Pojskic H., Zombra Ž., Washif J.A., Pagaduan J.C. (2023). Acute effects of loaded and unloaded whole-body vibration on Vertical Jump performance in Karate athletes. J. Hum. Kinet..

[8] Turner A.P., Sanderson M.F., Attwood L.A. (2011). The acute effect of different frequencies of whole-body vibration on countermovement jump performance. J. Strength Cond. Res..

[9] Lamont H.S., Cramer J.T., Bemben D.A., Shehab R.L., Anderson M.A., Bemben M.G. (2010). The acute effect of whole-body low-frequency vibration on countermovement vertical jump performance in college-aged men. J. Strength Cond. Res..

[10] Armstrong W.J., Grinnell D.C., Warren G.S. (2010). The acute effect of whole-body vibration on the vertical jump height. J. Strength Cond. Res..

[11] Horiuchi G., Nakashima H., Sakurai S. (2024). Mechanical energy flow in torso during baseball toss batting. Sports Biomech..

[12] Chen Y., Su Q., Yang J., Li G., Zhang S., Lv Y., Yu L. (2023). Effects of rest interval and training intensity on jumping performance: A systematic review and meta-analysis investigating post-activation performance enhancement. Front. Physiol..

[13] Abercromby A.F., Amonette W.E., Layne C.S., McFarlin B.K., Hinman M.R., Paloski W.H. (2007). Vibration exposure and biodynamic responses during whole-body vibration training. Med. Sci. Sports Exerc..

[14] Ae K., Koike S., Kawamura T. (2020). Kinetic function of the lower limbs during baseball tee-batting motion at different hitting-point heights. Sports Biomech..

[15] Ae K., Koike S., Fujii N., Ae M., Kawamura T. (2017). Kinetic analysis of the lower limbs in baseball tee batting. Sports Biomech..

[16] Punchihewa N.G., Miyazaki S., Chosa E., Yamako G. (2020). Efficacy of inertial measurement units in the evaluation of trunk and hand kinematics in baseball hitting. Sensors.

[17] Punchihewa N.G., Arakawa H., Chosa E., Yamako G. (2021). A hand-worn inertial measurement unit for detection of bat–ball impact during baseball hitting. Sensors.

[18] Kuo S.Y., Cheng K.B., Lee Y.C. (2021). Differences in baseball batting movement patterns between facing a pitcher and a pitching machine. Res. Q. Exerc. Sport.

[19] Katsumata H., Himi K., Ino T., Ogawa K., Matsumoto T. (2017). Coordination of hitting movement revealed in baseball tee-batting. J. Sports Sci..

[20] Takamido R., Yokoyama K., Yamamoto Y. (2022). Hitting movement patterns organized by different pitching movement speeds as advanced kinematic information. Hum. Mov. Sci..

[21] Tsutsui T., Sakata J., Sakamaki W., Maemichi T., Torii S. (2023). Longitudinal changes in youth baseball batting based on body rotation and separation. BMC Sports Sci. Med. Rehabil..

[22] Gray R. (2020). Changes in movement coordination associated with skill acquisition in baseball batting: Freezing/freeing degrees of freedom and functional variability. Front. Psychol..

[23] Kishita Y., Ueda H., Kashino M. (2020). Temporally coupled coordination of eye and body movements in baseball batting for a wide range of ball speeds. Front. Sports Act. Living.

[24] Layne C. (2007). Variation in neuromuscular responses during acute whole-body vibration exercise. Med. Sci. Sports Exerc..

[25] Chang C.Y., Hung M.H., Ho C.S., Lin K.C. (2019). The acute effects of whole-body vibration on fencers’ special abilities. Percept. Mot. Ski..

[26] Annino G., Iellamo F., Palazzo F., Fusco A., Lombardo M., Campoli F., Padua E. (2017). Acute changes in neuromuscular activity in vertical jump and flexibility after exposure to whole body vibration. Medicine.

[27] Blazevich A.J., Babault N. (2019). Post-activation potentiation versus post-activation performance enhancement in humans: Historical perspective, underlying mechanisms, and current issues. Front. Physiol..

[28] Boullosa D., Beato M., Iacono A.D., Cuenca-Fernández F., Doma K., Schumann M., Behm D.G. (2020). A new taxonomy for postactivation potentiation in sport. Int. J. Sports Physiol. Perform..

[29] Finlay M.J., Bridge C.A., Greig M., Page R.M. (2022). Upper-body post-activation performance enhancement for athletic performance: A systematic review with meta-analysis and recommendations for future research. Sports Med..

[30] Horiuchi G., Nakashima H. (2024). Relationship between ground reaction force in horizontal plane and mechanical energy flow in torso during baseball tee batting. Sports Biomech..

[31] Ae K., Burke D., Kawamura T., Koike S. (2024). Optimisation of the upper body motion for production of the bat-head speed in baseball batting. Sports Biomech..

[32] Horiuchi G., Nakashima H. (2024). Torso dynamics during follow through in baseball batting. Sports Biomech..

[33] Dowling B., Fleisig G.S. (2016). Kinematic comparison of baseball batting off of a tee among various competition levels. Sports Biomech..

[34] Escamilla R.F., Fleisig G.S., DeRenne C., Taylor M.K., Moorman C.T., Imamura R., Barakatt E., Andrews J.R. (2009). A comparison of age level on baseball hitting kinematics. J. Appl. Biomech..

[35] Yang C., Jin P. (2025). Factor analysis of the improvement of bat energy in baseball hitting. J. Hum. Sport Exerc..

[36] Prieske O., Behrens M., Chaabene H., Granacher U., Maffiuletti N.A. (2020). Time to differentiate postactivation “potentiation” from “performance enhancement” in the strength and conditioning community. Sports Med..

[37] Seitz L.B., Haff G.G. (2016). Factors modulating post-activation potentiation of jump, sprint, throw, and upper-body ballistic performances: A systematic review with meta-analysis. Sports Med..

[38] Krzysztofik M., Wilk M., Stastny P., Golas A. (2021). Post-activation performance enhancement in the bench press throw: A systematic review and meta-analysis. Front. Physiol..

[39] Muniz-Pardos B., Gómez-Bruton A., Matute-Llorente Á., González-Agüero A., Gómez-Cabello A., Casajús J.A., Vicente-Rodríguez G. (2020). Long-Term effects of whole-body vibration in trained adolescent swimmers: Does it increase strength, power, and swimming performance?. Int. J. Sports Physiol. Perform..

